# Adding Mobile Elements to Online Physical Activity Interventions Targeted at Adults Aged 50 Years and Older: Protocol for a Systematic Design

**DOI:** 10.2196/31677

**Published:** 2022-07-12

**Authors:** Eline H G M Collombon, Catherine A W Bolman, Denise A Peels, Gert-Jan de Bruijn, Renate H M de Groot, Lilian Lechner

**Affiliations:** 1 Faculty of Psychology Open Universiteit Heerlen Netherlands; 2 Amsterdam School of Communication Research University of Amsterdam Amsterdam Netherlands; 3 Department of Communication Science University of Antwerp Antwerp Belgium; 4 Faculty of Educational Sciences Open Universiteit Heerlen Netherlands

**Keywords:** mHealth, eHealth, physical activity, older adults, design protocol

## Abstract

**Background:**

Physical activity (PA) can increase mental and physical health in adults aged 50 years and older. However, it has been shown that PA guidelines are often not met within this population. Therefore, our research group developed 2 computer-tailored intervention programs in the last decade to stimulate PA: Active Plus and I Move. Although these programs were proven effective, positive effects diminished over time and attrition rates were relatively high. To respond to this, we will integrate 3 interactive mobile elements into the existing programs: activity tracker, ecological momentary intervention program, and virtual coach app.

**Objective:**

The goal of the research is to define systematic and evidence-based steps for extending our online computer-based PA intervention programs with 3 interactive mobile elements.

**Methods:**

Components often included in other (eHealth) design models were identified as key components and served as a base for the definition of systematic steps: exploration of context, involvement of the target population, prototype and intervention testing, and implementation. Based on these key components, 10 systematic steps were defined. The initial step is a literature search, with the results serving as a base for development of the low-fidelity prototypes in step 2. The pilot phase comprises the 3rd to 6th steps and includes semistructured interviews, pilot tests, and adaptations of the prototypes with intensive involvement of the target population of adults aged 50 years and older, where particular attention will be paid to lower educated persons. The 7th step is an effect evaluation in the form of a randomized controlled trial. During the 8th step, the most effective intervention programs will be selected and reinforced. These reinforced intervention programs will be used during the design of an implementation plan in the 9th step and the subsequent field study in the 10th step.

**Results:**

The project will be executed from December 2019 to December 2023. During this period, the systematic approach presented will be practically executed according to the methodological procedures described.

**Conclusions:**

Based on the 4 identified key components, we were able to design an evidence-based systematic design approach for separately adding 3 mobile elements to our existing online PA intervention programs. The 10 steps are presented as a useful approach to guide future eHealth design studies.

**International Registered Report Identifier (IRRID):**

DERR1-10.2196/31677

## Introduction

Stimulation of physical activity (PA) in adults aged over 50 years can result in health benefits, improved mood, an increase in self-esteem, and improved quality of life [1]. Furthermore, sufficient PA in adults aged over 50 years has been shown to help maintain physical and cognitive function thereby reducing the risk of falls and dementia, both major obstacles for retaining independence [2]. The World Health Organization recommends that adults engage in PA of moderate intensity for at least 150 minutes every week, spread over several days. In addition, bone and muscle strengthening activities are recommended at least 2 times per week, with older adults supplementing the regimen with balance exercises [3]. Globally, the trend is that older adults meet these guidelines less often since they engage in less PA than younger adults and this gap increases with age [4]. In addition, taking into account that the older population is growing faster than the total population in most regions of the world [5,6], it is clear that stimulation of PA among people aged over 50 years is of major relevance.

In the last decade, eHealth interventions, also known as digital health interventions, are emerging as a cost-effective and accessible method for PA promotion. It has been shown that such interventions are promising in increasing PA levels, especially when they are based on solid theory and use behavior change techniques that are evidence-based [7-9]. In recent years, our research group has developed several effective theory-based eHealth intervention programs for a variety of populations [10-14]. Relevant for this study are Active Plus and I Move. Active Plus is a web-based computer-tailored intervention program to promote PA among people aged older than 50 years [11,15]. Preceded by a questionnaire comprising questions on factors such as current PA levels and perceived PA beliefs and barriers, a computer-tailoring program generates and sends personalized advice, tips, and exercises based on these responses. Participants receive this tailored advice 3 times, where the information is based on the participant’s motivational stage of change, their motives and beliefs about being physically active, their self-efficacy levels, and the influence of their social environment [16]. The Active Plus intervention program is further based on the theory of planned behavior [17], social cognitive theory [18], and the health belief model [11,19]. On the contrary, I Move [10,20] is a more interactive and autonomy-supporting eHealth intervention program for adults based on the self-determination theory [21] and motivational interviewing [10,22]. I Move entails 4 automated text- and video-based sessions during which participants answer several questions. Since they receive directly tailored feedback messages based on the answers of these questions, a motivational dialogue is simulated between the intervention program and the participant [10]. Participants are recommended to follow Active Plus and I Move via a computer, laptop, or tablet, as it is not suitable to be used on a smartphone. Both these intervention programs were systematically developed using the intervention mapping (IM) protocol [23].

Although Active Plus and I Move have been proven effective in increasing levels of PA in the short term [15,20], these positive intervention effects decreased when follow-up time increased, which is in line with conclusions of meta-analyses [24,25]. However, maintenance of behavioral intervention effects is of major importance to achieve an impact on public health [26]. One possible explanation for the decrease in effectiveness can be found in the high attrition rates often seen in studies investigating the effects of eHealth interventions [27].

Besides computer-based eHealth interventions, mobile technologies known as mHealth have recently emerged as another promising method for stimulation of PA. Several studies have already proved the effectiveness of mobile technologies in stimulating PA in a variety of populations [28-33]. These positive effects can be explained by the increasing use of smartphones among all populations and, as a result, a more pronounced just-in-time and interactive nature of mobile technologies compared to the less flexible and in-time computer-based technologies. Although PA intervention programs including both computer and mobile technologies are emerging in recent years, they are still less common compared to intervention programs where only one of the technologies is used. Based on earlier research, it can be expected that eHealth and mHealth technologies reinforce each other when they are combined within one PA intervention program [34]. As a result, both short-term and long-term intervention effects and user engagement are expected to increase when compared to an intervention where only one of the methods is used. The mHealth technologies have several advantages such as just-in-time information, interactivity, and adaptiveness [35].

One promising mHealth technology is an activity tracker, which incorporates elements for self-monitoring, goal-setting, and feedback and have been shown to be an effective tool for increasing PA [32,36]. Effectiveness is further increased when combined with a mobile app, giving more detailed readily available feedback on a larger screen compared with the screen of the tracker [36]. Advantages of these trackers are that they enable objective measurements of PA behavior, passive data gathering without the need of active input of the participant, and the possibility to provide just-in-time tailored feedback on PA (eg, on the number of steps taken that day) [37,38]. Importantly, earlier research has shown that older adults are willing to use this technology [39-41].

Second, ecological momentary interventions (EMI) have emerged in recent years to stimulate PA. Within an EMI program, short questionnaires are send to a participant during the day to investigate their personal situation at that moment. Based on the answers, a tailored PA message that takes into account the current personal situation of a participant can be delivered. The benefit of such programs is that they can deliver just-in-time tailored messages to create self-awareness and provide strategies for being physically active. As a result, they can deliver feedback when a difficult situation occurs and give tips to overcome barriers or avoid risks related to PA [42,43]. In contrast to the passive data collection of activity trackers, EMI demands a more active contribution from a participant to get insight in relevant situations or moods that may relate to PA behavior, since they are asked to complete short questionnaires several times per day. The delivery of these questionnaires is known as ecological momentary assessment. To our knowledge, not much research has been done regarding the use and acceptance of smartphone-based EMI programs for PA promotion in the population of adults aged over 50 years. However, the study by King et al [44] showed promising results regarding the use and acceptance of handheld computers to promote PA in underactive older adults.

Furthermore, interactive virtual coach apps (using chatbots) are promising technologies to improve PA behavior [45]. A chatbot delivers persuasive tailored PA chat messages via a smartphone app to participants throughout the day. Message selection can be based on variables like step count measured via an app and machine learning algorithms [45,46]. These algorithms adaptively learn which message will be the most persuasive, given the specific context and preferences of an individual and taking into account previous responses to the messages. A possible benefit of this app compared with activity trackers and EMI is the ability to calculate and deliver the most effective, adaptive, tailored, and persuasive messages in an unobtrusive and familiar way at specific time points throughout the day without any active input from the user.

During this study, our existing computer-based intervention programs Active Plus and I Move will be enriched with 1 of 3 previously mentioned mobile-based elements, either an activity tracker, EMI program, or virtual coach app (using a chatbot). This will result in 3 new versions of both Active Plus and I Move. The use of a systematic approach for the renewal of the intervention programs is considered essential since this contributes to the preservation of the proven effectiveness of the Active Plus [15] and I Move [20] intervention programs. In recent years, several systematic design models applicable to eHealth and mHealth intervention development were presented in the literature [23,47,48]. In particular, intervention mapping, used in the development of Active Plus and I Move, is frequently applied [23]. The aim of our study is to add the mobile elements separately on top of the existing, retained, and IM-based Active Plus and I Move intervention programs. As a result, we are building on the previous IM results during the integration of mobile elements with the existing online PA intervention programs. To retain the effectiveness of the existing programs, the use of a systematic design approach was considered essential. Therefore, the aim of this study was to define systematic and evidence-based steps for integrating the 3 mobile elements with the computer-based Active Plus and I Move intervention programs based on the combined insights of earlier presented design models and protocols. The aim of this paper is to present the defined systematic design steps and the associated methodological procedures.

## Methods

### Defining the Steps of the Systematic Approach

#### Identification of Key Components

To define the design steps of our systematic approach, we used several existing models and protocols as a base. Examples are the more general IM protocol as well as models specifically for the development of eHealth and mHealth interventions, such as the spiral technology action research model, the CeHRes (Center for eHealth Research and Disease Management) roadmap [37], and the behavioral intervention technology model [49,50].

Although these models differ regarding the number of steps included and specific objective, key components recur in several models and can be identified. These key components served as a base for the subsequent definition of the systematic design steps for integration of the mobile elements with the existing online intervention programs within this study.

#### Exploration of Context

Exploration of context, where relevant information related to the topic is collected, was identified as an important initial step for a design process prior to starting the development of the first prototypes [23,37,51]. eHealth design studies often refer to a preparatory phase where a literature study is performed, the expertise of professionals is used, and the preferences of the target population are assessed [52,53]. Although these elements are mainly included during a preparatory phase, it is important to keep up with published evidence throughout the process, since it can alert developers to issues that might impact continuation of the development process [54]. This might lead to different (intervention) strategies.

#### Involvement of the Target Population

Involvement of the target population, also known as participation [23,55], is considered the key component for eHealth design and is therefore included in the design approach presented here. Research shows that when beneficiaries are involved in the design and dissemination of online health interventions and elements, the outcomes are more likely to be successful [56,57]. Additionally, it has been shown that older adults interact differently with information technology compared to younger people [58,59]. Therefore, interviews, focus groups, and pilot tests among the target population could be valuable to include in a design process. With our initial eHealth intervention programs, we are aiming to reach all people aged 50 years and older, regardless of gender, level of education, socioeconomic status, and activity level. Since it has been shown that digital interventions are less often used by vulnerable older adults with low education and low eHealth literacy [60], the focus should be on the preferences of this population to improve accessibility. By involving the target population (especially laggards in the use of digital apps) in the design process, higher rates of usability are expected, defined as “the extent to which a user can use a product to achieve specific goals with effectiveness, efficiency, and satisfaction” [61].

#### Prototype and Intervention Testing

Another important component during an eHealth design process is intervention testing [51]. First, iterative cycles of pilot testing of prototypes contribute to the improvement of the intervention. Additionally, low-fidelity prototypes could be tested during interviews and focus groups among the target population to provide visualization of the ideas, elicit preferences and requirements, and support the creative process [52]. Finally, the effects of the developed interventions should be evaluated in a large-scale [23] randomized controlled trial (RCT) [51,62].

#### Implementation

Evaluation of a new intervention in a research setting is not the end point of a development process. After showing the effectiveness of an experiment, it is important that the intervention is implemented in practice [54]. A detailed plan summarizing the factors that facilitate or impede implementation is needed to embed the new intervention in practice and overcome the research-practice gap. During development of this implementation plan, the use of validated tools, such as the IM protocol [23], eHealth implementation toolkit [63], readiness for implementation model [64], and NASSS framework [65], could be considered to increase the odds of success.

#### Steps in the Systematic Approach

**Figure 1:**
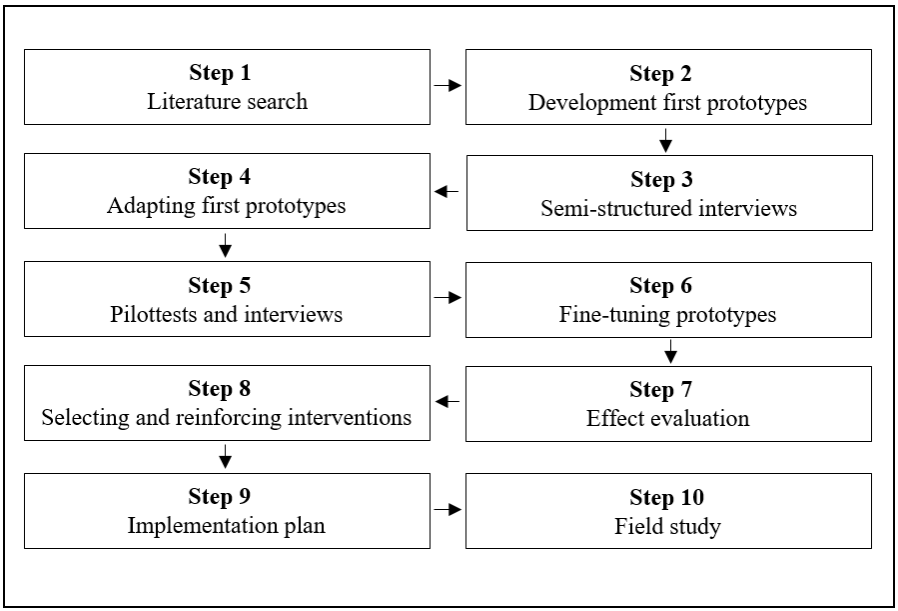
Overview of design steps.

The 4 key components were used as a base for defining the steps of our systematic approach: (1) exploration of context, (2) involvement of the target population, (3) prototype and intervention testing, and (4) implementation. Based on these key components, in combination with the more traditional steps in systematic intervention development, 10 evidence-based steps for extending our online PA intervention programs with mobile elements were defined. Figure 1 provides a schematic overview of the design steps.

#### Step 1: Literature Search

Literature searches will be performed per additional mobile element. For all 3 elements, the existing literature on attitude, usefulness, and ease of use regarding the mobile element within our target population of adults aged over 50 years will be searched. Additionally, for the activity tracker, we will investigate whether specific design features and preferences need to be taken into account for this population during selection of an appropriate tracker. This will be complemented with a commercial market study to select appropriate devices that match the earlier identified design features and preferences. For the EMI element, the existing literature regarding barriers and motivators for adults aged over 50 years to participate in PA will be searched to serve as a base for development of the ecological momentary assessment questionnaire and the EMI messages. Additionally, earlier published studies related to EMI interventions will be investigated on relevant design guidelines for the development of our own program. For the chatbot element, an already existing app originally developed for the Supreme Nudge project [45] will be used as a starting point. This chatbot consists of 2 apps, a step count app and a chat app to deliver the persuasive messages. Literature regarding this project will be thoroughly searched and a more general literature search on chatbots in relation to PA will be conducted. Last, a literature search will be conducted to acquire more knowledge on particular design guidelines to reach adults aged over 50 years with lower levels of education and low eHealth literacy or digital skills.

#### Step 2: Development of First Prototypes

Based on the results of the literature search, the additional mobile elements will be designed and subsequently integrated with both Active Plus and I Move. To secure the privacy of users, a detailed data management plan based on the General Data Protection Regulation was prepared prior to the start of our study and will be followed during the complete practical execution of the design approach.

For the activity tracker, the literature search comprises among other things the selection of an appropriate tracker; costs will be considered due to attainable future implementation. The results of the literature search regarding EMI will be used to choose an appropriate technical format and protocol to deliver the prompts, develop an assessment questionnaire, and identify relevant topics for the advisory intervention messages. An already existing chatbot comprising step count and chat apps [45] will be adapted to fit into the current online PA intervention programs. Messages related to manually mapped GPS locations will, for example, be replaced by location-based weather messages in order to enable a recruitment procedure at a national level at later stages of the study.

**Figure 2:**
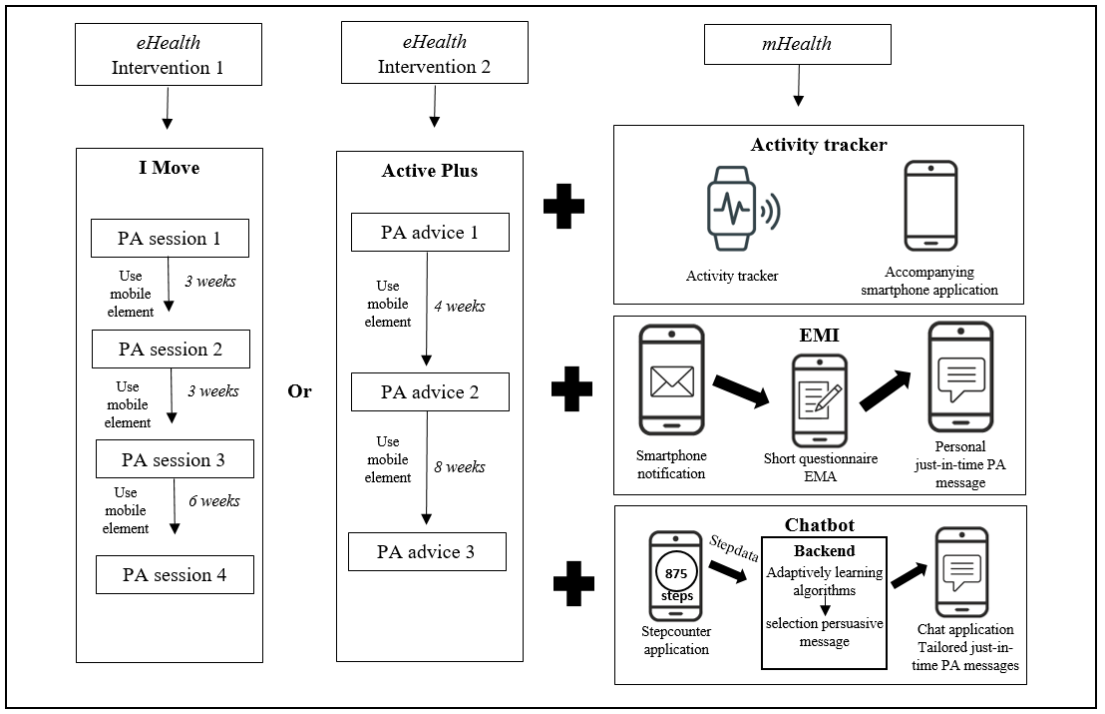
Overview of interventions. EMA: ecological momentary assessment; EMI: ecological momentary intervention; mHealth: mobile health; PA: physical activity.

For all elements, linked components between the mobile element and the existing online PA intervention programs will be designed to improve the degree of interplay. An example of this interplay is the addition of advice related to the mobile element within the intervention programs. Furthermore, information and instruction manuals will be developed based on the guidelines for lower-literate users resulting from the literature search. In addition to the results of the literature search, software capabilities and privacy guidelines will be considered during the development of the prototypes. In the end, this will result in 3 extended low-fidelity prototype versions per eHealth intervention program: (1) Active Plus or I Move including activity tracker, (2) Active Plus or I Move including EMI, and (3) Active Plus or I Move including chatbot. An overview of the different mobile elements and online intervention programs is shown in Figure 2.

#### Steps 3 and 4: Semistructured Interviews and Adapting First Prototypes

The next step is the organization of interviews among adults aged over 50 years. Thus, from this step on, the target population will be intensively involved in the design process. The aim is to include a sample of adults aged over 50 years that varies by characteristics such as level of education, age, gender, PA levels, and digital skills. The purpose of the interviews is to improve usability and acceptability of the low-fidelity prototypes for the target population. Participants will test parts of the prototypes and answer questions based on a semistructured interview protocol. Topics such as usability, ease of use, attitude, (privacy related) concerns, preferences, capabilities and needs regarding the mobile elements, and the combination with Active Plus and I Move will be covered. During development of the semistructured interview protocol, validated tools such as the System Usability Scale [66] and theoretical models such as the technology acceptance model [67] will be used as guidelines. The results of the interviews will be used to further refine the set of core components based on the needs of the target population and improve the low-fidelity prototypes of the updated versions of Active Plus and I Move.

#### Steps 5 and 6: Pilot Tests, Interviews, and Fine-tuned Prototypes

The adapted prototypes of the 3 new versions each of Active Plus and I Move will be pilot-tested among the target population of adults aged over 50 years. Participants will be recruited via social media advertisements and after registration equally divided among the following research groups (n=10 per group): (1) Active Plus including activity tracker, (2) Active Plus including EMI, (3) Active Plus including chatbot, (4) I Move including activity tracker, (5) I Move including EMI, and (6) I Move including chatbot. The original intervention programs have a duration of 12 weeks, but for the pilot test, a shortened 2-week version with a focus on the interplay between the online PA intervention program and the mobile element will be used.

After registration, participants will receive an information package comprising an information letter, instructions for the mobile element, and a daily testing diary. Additionally, participants allocated to the activity tracker groups will receive a tracker with the information package. No additional materials beyond the instruction manuals are needed for the EMI or chatbot element. Participants will complete the computer-based baseline questionnaire (T0). Participants will then gain access to the first online advice session of either Active Plus or I Move. During the advice session, they will receive information and instructions regarding the added mobile element. They will then use the assigned element for 2 weeks and complete a daily entry in the testing diary (Multimedia Appendix 1). After the 2 weeks, participants will be invited via email to complete the second online advice session. This advice includes advisory texts focused on the mobile element they received. For example, for the activity tracker and chatbot groups, additional information on step count will be provided, since both elements are able to measure this. After the second advice session, participants will complete a more extensive questionnaire investigating effects, usability, and acceptability of the intervention program and the added mobile element (Multimedia Appendix 2). This questionnaire will be composed based on validated tools such as the System Usability Scale [66] and theoretical models such as the technology acceptance model [67]. After the pilot tests, a sample of participants (who provided consent) will be invited for an interview to gather qualitative in-depth information regarding their experiences. For these interviews, semistructured discussion guides will be developed specifically for the assigned mobile element.

#### Step 7: Effect Evaluation

During the seventh step of the design process, the effects and usability of the extended intervention programs will be evaluated by means of an RCT. The trial consists of 3 experimental conditions and one waitlist control group. According to our sample calculation (effect size=0.3; β=0.8) and taking into account a commonly reported attrition rate of 40% within eHealth studies [15,20], 200 participants will be included per arm. The following experimental conditions will be tested: (1) online PA intervention program including activity tracker, (2) online PA intervention program including EMI, and (3) online PA intervention program including chatbot. Within these conditions, there are 2 subconditions: Active Plus (n=100) and I Move (n=100). Eligible participants are 50 years or older; able to use a computer, laptop, or tablet; and have a smartphone. The aim is to have a varied research group in terms of gender, age, level of education, etc. In order to reach a varied sample of participants, a detailed recruitment plan will be made prior to the trial.

Interested people who meet the inclusion criteria can register via a website where they sign an online informed consent and enter some personal details. Subsequently, automatic randomization will take place within the software of the online PA intervention programs. First, an accelerometer (GT3X-BT, ActiGraph LLC) with instructions and a return envelope will be sent to participants to gain insight in their current PA behavior. Participants are instructed to wear the accelerometer for 7 days. Around the seventh and last day of wearing the accelerometer, participants will receive an information package via post that includes for all groups a more specific information letter and credentials for Active Plus or I Move. Additionally for the experimental groups, materials needed for the assigned mobile element are included. Participants are instructed to complete the baseline questionnaire T0 after finishing the 7-day accelerometer wear period. This questionnaire can be accessed by logging in with the credentials for either Active Plus or I Move. Subsequently, participants in the experimental conditions will follow the intervention programs, which have a total duration of 12 weeks. All research groups will complete follow-up questionnaires 3 months (T1) and 6 months (T2) after baseline. The week before questionnaire T2, participants will again receive an accelerometer via post with instructions to wear it again during a preset period of 7 days. The waitlist control group will receive the Active Plus advice combined in 1 advice after completion of the last measurement (T2).

**Figure 3:**
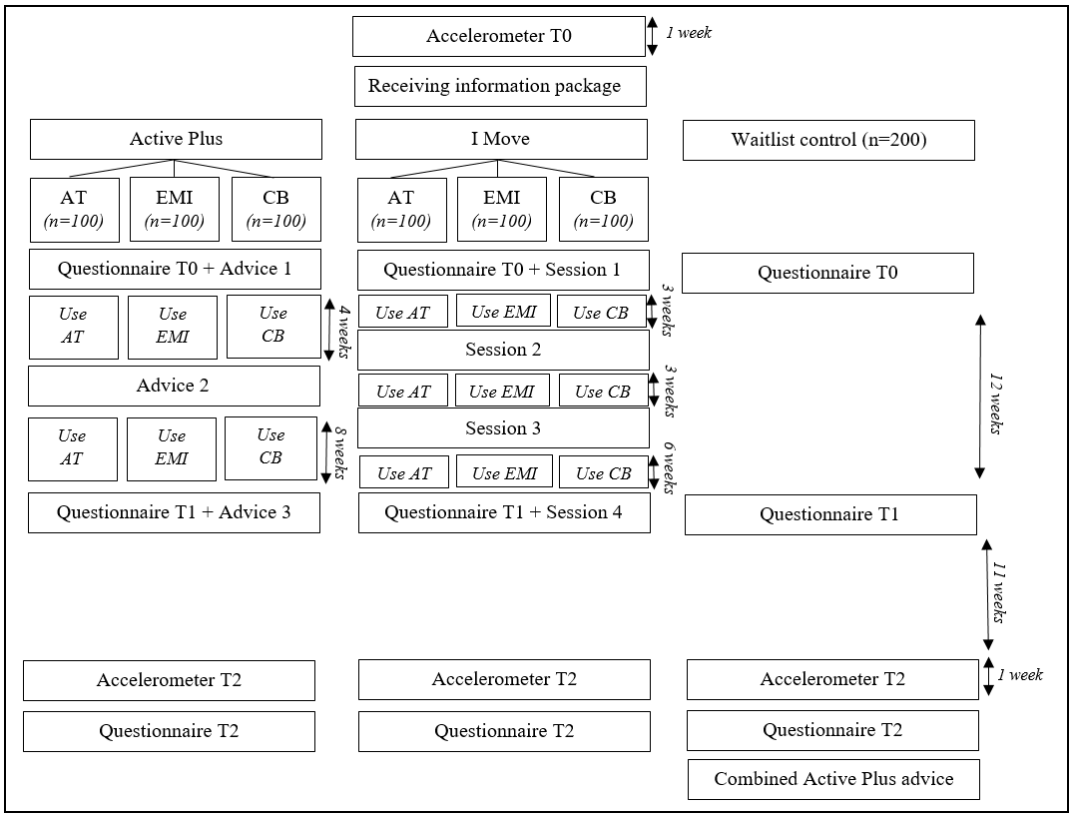
Schedule of procedures. AT: activity tracker; CB: chatbot; EMI: ecological momentary intervention

The primary outcome will be PA behavior, which will be subjectively assessed via the validated Short Questionnaire to Assess Health-Enhancing Physical Activity (SQUASH) [68] at T0, T1, and T2 and objectively measured with an accelerometer at T0 and T2. Secondary outcomes, measured at T0, T1, and T2, will be intention to be physically active, commitment toward being physically active, and self-efficacy related to PA. Additionally, factors such as usability of and engagement with the interventions and specifically the mobile elements will be tested in the experimental conditions using evaluation questionnaires during T1 and T2 (Multimedia Appendix 3). Examples of questions are “I would like to continue using the activity tracker/EMI/chatbot” (5-point scale: 1=completely disagree to 5=completely agree), “What improvements can be made to the program you have followed?” (open question), and “How much fun did you have using the activity tracker/EMI/chatbot?” (1-10 rating). Last, use of the interventions and dropout of participants will be assessed based on process evaluation data. An overview of the research design of the RCT is shown in Figure 3.

#### Step 8: Selecting and Reinforcing the Interventions

During a data science–oriented parallel study of the project, the most effective components of previous online PA interventions (eg, Active Plus and I Move) without the added mobile elements are identified by using Bayesian network analyses. For these analyses, 8 large-scale existing data sets from 5 proven effective online interventions to stimulate PA (N>5000), developed and conducted by our research group, will be merged into an integrated data set and analyzed [15,20,69-71]. This will provide knowledge on which relevant demographic factors (eg, age, gender, education), determinants of PA, and behavior change techniques are most relevant to increase intervention use and PA among adults and older adults to enhance both effect sizes and effectiveness of online computer-tailored PA intervention programs. More detailed information regarding these data analyses and preliminary results is published elsewhere [72]. The most suitable mobile element resulting from the effect evaluation of step 7 will then be added to the strengthened intervention programs. These reinforced intervention programs will be pilot-tested for effects, usability, and acceptability (n=30).

#### Step 9: Implementation Plan

In the ninth step, a detailed implementation and dissemination plan will be written for using the reinforced intervention programs in practice. This is a preparatory phase for the field study in the tenth step. Several steps are included during the development of this implementation plan according to the implementation mapping protocol [73] and the NASSS (nonadoption, abandonment, scale-up, spread, sustainability) framework [65]. Program adopters and implementers are already identified since they are part of our consortium. In cooperation with these already identified adopters and implementers, more potentially relevant stakeholders will be identified. Their needs and perceived barriers and facilitators regarding the implementation will then be assessed via interviews and group sessions. Based on the insights gained, appropriate previously proven effective implementation strategies will be selected [74] and a detailed implementation plan will be developed. As a result, it is expected that as the feasibility in practice improves, the facilitators of adoption are better embedded for use of the intervention programs in practice [75,76], and the research-practice gap diminishes.

#### Step 10: Field Study

The 2 reinforced intervention programs will be tested and implemented in practice according to the implementation plan created in step 9. Both interventions will be tested (n=200 per intervention) with main assessments in the form of questionnaires at baseline (T0), 3 months postbaseline (T1), and 6 months postbaseline (T2). Factors such as PA (SQUASH [68]), intention to participate in PA, and PA-related self-efficacy will be assessed. The aim is to provide insight into whether the adaptations result in a practical setting in increased use of the interventions, PA levels, and maintenance of PA levels and in decreased dropout compared to the original online interventions without mHealth apps. Since the main focus is on implementation in the field, no control groups will be included during this phase. The results will be compared with the already available detailed data on use of the original Active Plus and I Move intervention programs and effects and effect sizes of previous versions of the intervention programs. As a result, studying whether use and effects have improved and whether dropout has decreased is still possible. Again, a strong focus will be on vulnerable populations such as the lower educated and those with low eHealth skills during this field study.

Additionally, attention will be paid to factors such as data infrastructure and data management in relation to implementation in practice and whether additional instruction or training for intermediaries or end users is needed. At the end of the field study, a short process and dissemination evaluation will take place based on the data of this quantitative study combined with interviews with stakeholders and end users. The aim of this part of the evaluation is to gain insight into ways to sustain the reinforced intervention programs in practice with an emphasis on the facilitating and impeding factors for broadscale implementation.

#### Ethics Approval

All aforementioned procedures of steps 1 to 10 of the systematic approach will be approved by the central ethical review committee of the Open Universiteit. Additionally, all data will be obtained and stored according to the composed data management plan and following the general data protection regulation.

## Results

Funding for this study was provided by grant 546003005 (ZonMW) from The Netherlands Organization for Health Research and Development. The project will be executed from December 2019 to December 2023. During this period, the systematic approach presented here will be practically executed according to the described methodological procedures.

## Discussion

### Aim of the Study

The aim of this study was to define a systematic and evidence-based approach for separately integrating 3 mobile elements with the computer-based Active Plus and I Move intervention programs based on the combined insights of design models and protocols presented earlier. Based on 4 identified key components, which resulted from an analysis of existing eHealth design models in combination with the more traditional intervention design models, we were able to compose 10 systematic design steps to guide the development process.

### Strengths and Limitations

Use of these systematic steps for extending our online PA intervention programs with mobile elements is considered a strength of this study and essential for various reasons. First, it is important to retain the already proven effectiveness [15,20] of the original computer-based PA intervention programs Active Plus and I Move. Second, optimal and iterative involvement of the target population during the design process is effectuated since attention is paid to this repeatedly at each step. Last, results from a previous step are often used as input for the next step. As a result, data analysis takes place more gradually during the design process instead of only after finishing the development of the new intervention elements and the complete design study. Therefore, interim (prototype) intervention adjustments are possible which will contribute to a better end product. Following the design steps presented in this study might be useful for future eHealth and mHealth design studies since it is an evidence-based systematic development and evaluation approach.

Although, it is clear that the use of a systematic design approach is essential for successful intervention development, clear and thorough descriptions of the prior development process of online and mobile health interventions are often lacking. This impedes research and intervention development, as eHealth developers often start from scratch when creating or adapting an online intervention or mobile element. Therefore, more publications extensively describing the followed design process leading to a new eHealth intervention or mHealth element are warranted. By describing the followed steps for the separate integration of 3 mobile elements with our existing online PA intervention programs in this study, we aimed to contribute to this.

A possible limitation of this study is that the systematic design approach will only be executed once in practice. The design approach could be lifted to a higher level by applying iterative cycles and processes according to the CeHRes model [48]. For example after fine-tuning the prototypes in step 6, there is an option to return to step 5 and perform a second pilot test with the fine-tuned prototype. Increasing the number of iterative cycles may result in higher levels of usability, satisfaction, and acceptability of the interventions [77].

By separately integrating an activity tracker, EMI program, and chatbot with our already effective proven online intervention programs according to the designed systematic approach, the positive short-term effects on PA may be further enhanced and may be better maintained in the longer term. Additionally, engagement with the intervention programs may increase and attrition may decrease. A potential strength of adding mobile elements to our existing online PA intervention programs is that participants are expected to be more actively involved on a daily basis with the intervention [78], so levels of boredom may decrease and attention may increase [79]. Furthermore, by following a systematic design approach with involvement of the target population, levels of usability and acceptability of the renewed interventions will possibly increase [56,57], which might be a predictor for engagement [80]. However, the results of the RCT will provide insight whether the addition of mobile elements to our online PA intervention programs indeed increase the effectiveness of and engagement with our interventions, whether attrition rates decrease, and which mobile element scores best on factors such as usability and practical applicability. Based on these results, decisions will be made regarding the intervention programs that will be used in practice during the implementation phase of the study. Extensive results of the practical execution of the systematic design steps will be described in separate articles.

### Conclusion

In conclusion, based on the 4 key components identified, we were able to design an evidence-based systematic approach for separately adding 3 mobile elements to our existing online PA intervention programs. The 10 systematic design steps of this approach and the associated methodological procedures are presented in this paper. The systematic steps are presented as a useful approach to guide future eHealth and mHealth design studies.

## Appendix

Multimedia Appendix 1 Example day 1 questionnaire testing diary Activity Tracker pilot test.

Multimedia Appendix 2 T1 questionnaire pilot test with the Active Plus including Chatbot version used as an example.

Multimedia Appendix 3 T1 questionnaire regarding mobile element and T2 questionnaire regarding combination intervention program and mobile element randomized controlled trial with the I Move including Activity Tracker version used as an example.

## Abbreviations

CeHRes: Center for eHealth Research and Disease Management
EMI: ecological momentary intervention
IM: intervention mapping
mHealth: mobile health technologies
NASSS: nonadoption, abandonment, scale-up, spread, sustainability
PA: physical activity
RCT: randomized controlled trial
SQUASH: short questionnaire to assess health-enhancing physical activity
