# Supplementary material for: Adding Mobile Elements to Online Physical Activity Interventions Targeted at Adults Aged 50 Years and Older: Protocol for a Systematic Design
Source: JMIR Res Protoc. 2022 Jul 12;11(7):e31677. doi: 10.2196/31677 (PMC9328785; doi:10.2196/31677)
Supplement: Multimedia Appendix 1 [file resprot_v11i7e31677_app1.pdf]

Day 1[1,2] **Date:___ / ___ / ________**

1) Did you wear your activity tracker today?	
☐ Yes, all day	*(Go to question 2a)*
☐ Yes, part of the day	*(Go to question 2b)*
☐ No	*(Go to question 2c)*

2a) Enter below why you have worn the activity tracker all day:

2b) Enter below why you have worn the activity tracker only a part of the day:

2c) Enter below why you haven't worn the activity tracker today:

3) Did you have any problems while using the activity tracker today?	
☐ Yes	(Go to question 4)
☐ No	(Go to question 5)

Pay attention! The questionnaire continues on the next page!

4) Enter below which problems you have had with the activity tracker today:

5) To what extent has the activity tracker helped you to be (more) physically active today?<br><br>*Please indicate a number from 1 to 10. Circle the number that fits your situation.*
1 = No help at all, 10 = helped a lot<br><br>**1 2 3 4 5 6 7 8 9 10**

6) How much fun did you have using the activity tracker today?<br><br>*Please indicate a number from 1 to 10. Circle the number that fits your situation.*
1 = no fun at all, 10 = a lot of fun<br><br>**1 2 3 4 5 6 7 8 9 10**

7) Please enter below what thoughts and/or experiences you had while using the activity tracker:

End of this questionnaire – Thank you for completing!

[1] *Activity tracker testing diary is presented here as an example.*
[2] *Testing diary was originally in Dutch and translated to English for this multimedia appendix.*
