# Supplementary material for: Adding Mobile Elements to Online Physical Activity Interventions Targeted at Adults Aged 50 Years and Older: Protocol for a Systematic Design
Source: JMIR Res Protoc. 2022 Jul 12;11(7):e31677. doi: 10.2196/31677 (PMC9328785; doi:10.2196/31677)
Supplement: Multimedia Appendix 2 [file resprot_v11i7e31677_app2.pdf]

T1 questionnaire pilottest – Active Plus including Chatbot example[1-3]

Questions 1 – 10 → validated System Usability Scale questionnaire

Additional author-developed questions:

11. Guided by the instructions, I was able to prepare the chatbot for use.
□ Completely disagree<br>□ Disagree<br>□ Disagree / agree<br>□ Agree<br>□ Completely agree

12. The chatbot helped me to be more physically active.
□ Completely disagree<br>□ Disagree<br>□ Disagree / agree<br>□ Agree<br>□ Completely agree

13. The chatbot and the advices provided via the Active Plus website worked well together.
□ Completely disagree<br>□ Disagree<br>□ Disagree / agree<br>□ Agree<br>□ Completely agree

14. The chatbot is a good addition to the advices provided via the Active Plus website.
□ Completely disagree<br>□ Disagree<br>□ Disagree / agree<br>□ Agree<br>□ Completely agree

15. How much fun did you have while using the chatbot?<br>*Please enter a rating on a scale from 1-10.*<br>*1 = no fun at all, 10 = a lot of fun*
**1 2 3 4 5 6 7 8 9 10**

16. How satisfied are you with the chatbot?<br>*Please enter a rating on a scale from 1-10.*<br>*1 = not satisfied at all, 10 = completely satisfied*
**1 2 3 4 5 6 7 8 9 10**

[1] Only author-developed questions (based on the referred validated tools) are presented here.
[2] Questions were asked online via the intervention program software with different lay-out than presented here.
[3] Questions were originally in Dutch and translated to English for this multimedia appendix.
