# Supplementary material for: Adding Mobile Elements to Online Physical Activity Interventions Targeted at Adults Aged 50 Years and Older: Protocol for a Systematic Design
Source: JMIR Res Protoc. 2022 Jul 12;11(7):e31677. doi: 10.2196/31677 (PMC9328785; doi:10.2196/31677)
Supplement: Multimedia Appendix 3 [file resprot_v11i7e31677_app3.pdf]

T1 questionnaire regarding mobile elements RCT – Example I Move including Activity Tracker[1,2]

1. I would like to continue using the activity tracker.
□ Completely disagree<br>□ Disagree<br>□ Disagree / agree<br>□ Agree<br>□ Completely agree

2. The activity tracker was easy to use.
□ Completely disagree<br>□ Disagree<br>□ Disagree / agree<br>□ Agree<br>□ Completely agree

3. With the accompanied instructions, I was able to use the activity tracker properly.
□ Completely disagree<br>□ Disagree<br>□ Disagree / agree<br>□ Agree<br>□ Completely agree

4. The activity tracker motivated me to be physically active.
□ Completely disagree<br>□ Disagree<br>□ Disagree / agree<br>□ Agree<br>□ Completely agree

5. How much fun did you have while using the activity tracker?<br>*Please enter a rating on a scale from 1-10.*<br>*1 = no fun at all, 10 = a lot of fun*
**1 2 3 4 5 6 7 8 9 10**

6. How satisfied are you with the activity tracker?<br>*Please enter a rating on a scale from 1-10.*<br>*1 = not satisfied at all, 10 = completely satisfied*
**1 2 3 4 5 6 7 8 9 10**

T2 questionnaire regarding intervention program + mobile element RCT – Example I Move including Activity Tracker[1,2]

1. The activity tracker and the online sessions of the program worked well together.
□ Completely disagree<br>□ Disagree<br>□ Disagree / agree<br>□ Agree<br>□ Completely agree

2. The activity tracker is a good addition to the online sessions of the program.
□ Completely disagree<br>□ Disagree<br>□ Disagree / agree<br>□ Agree<br>□ Completely agree

3. What grade do you give to the followed program (online sessions + activity tracker)?
**1 2 3 4 5 6 7 8 9 10**

4. Which improvements can be made to the program?<br>*You can enter more than one improvement.*

5. Did you encounter any technical problems while using the activity tracker? If yes, which one?

6. Which activity tracker did you use during the study?
□ The activity tracker received for the study<br>□ My own activity tracker / smartwatch<br>□ I didn’t use an activity tracker

[1] *Questions were asked online via the intervention program software with different lay-out than presented here.*
[2] *Questions were originally in Dutch and translated to English for this multimedia appendix.*
